# Validation of Bengali perceived stress scale among LGBT population

**DOI:** 10.1186/s12888-017-1482-0

**Published:** 2017-08-29

**Authors:** Muhammad Kamruzzaman Mozumder

**Affiliations:** Department of Clinical Psychology, University of Dhaka, Dhaka, 1000 Bangladesh

**Keywords:** Perceived stress scale, Factor analysis, CFA, EFA, LGBT, Bangladesh

## Abstract

**Background:**

Lesbian, gay, bisexual and transgender (LGBT) population encounter more stressful life circumstances compared to general population. Perceived Stress Scale (PSS) can be a useful tool for measuring their stress. However, psychometric properties of PSS have never been tested on LGBT population.

**Methods:**

This cross sectional study employed a two-stage sampling strategy to collect data from 296 LGBT participants from six divisional districts of Bangladesh. Exploratory (EFA) and confirmatory factor analysis (CFA) were carried out on PSS 10 along with analysis of reliability and validity.

**Results:**

EFA revealed a two-factor structure of PSS for LGBT population explaining 43.55% - 51.45% of total variance. This measurement model was supported by multiple fit indices during CFA. Acceptable Cronbach’s alpha indicated internal consistency reliability and high correlations with Self Reporting Questionnaire 20 demonstrated construct validity of PSS 10 for LGBT population.

**Conclusion:**

This study provided evidence of satisfactory psychometric properties of Bengali PSS 10 in terms of factor structure, internal consistency and validity among LGBT population.

**Electronic supplementary material:**

The online version of this article (10.1186/s12888-017-1482-0) contains supplementary material, which is available to authorized users.

## Background

Stress can be regarded as one of the most influential construct in understanding health and wellbeing. A major shift in conceptualizing stress occurred when Lazarus [1] pointed out the importance of individual’s appraisal and its interaction with environmental events in understanding stress. Inclination towards measurement of appraisal of stress rather than stressful life event itself has since been targeted. Cohen et al. [2] developed Perceived Stress Scale (PSS) to measure the degree to which an individual appraises his/her life situations as stressful. This appraisal based scale has been demonstrated to outperform life event based measures in assessment of stress [3]. Three different versions of PSS with 14, 10 and 4 items have been developed, and validated.

With evidence of sufficient validity and reliability indicated by studies conducted over different cultures and populations all over the world, PSS has been established as a robust tool for measuring perceived stress. PSS 10 demonstrated a stable two-factor structure where items 1, 2, 3, 6, 9 & 10 constitute the first factor and the remaining four items constitute the second factor [4–9]. The first factor is often termed as ‘general stressors’ [10], ‘perceived helplessness’ [11] or ‘negative perception’ [12]. The second factor is termed as ‘ability to cope’ [10], ‘perceived self-efficacy’ [11] or as ‘positive perception’ [12]. PSS has been translated and validated in more than 30 languages and on different populations including, general people [13], pregnant women [7], elderly workers [14], cancer patients [15], and psychiatric patients [16].

Compared to general population, lesbian, gay, bisexual and transgender (LGBT) people are more prone to experience stressful life events including stigma, discrimination, financial hardship, abuse (physical, sexual & psychological), and legal difficulties, [17–19]. Section 377 of Bangladesh penal codes clearly prohibits carnal intercourse in the ground of being “against the order of nature”. This same principle may be used against all variations of sexuality. However, the stress faced by LGBT population stems more from socio-cultural and religious rather than legal aspects. Mental health impact of minority stress among LGBT has been established for a long time [20, 21]. Studying stress among these sexual minority populations is essential. To ensure accurate interpretation and usability of research findings, contextually validated tools are required. Perceived Stress Scale can be a suitable option to measure stress among LGBT population. Although developed primarily on general population sample, its robustness in measuring stress among different population subgroups has been widely demonstrated [7, 14–16, 34]. The generic items of the PSS seem to fit well with appraisal of any stressors including the circumstances of LGBT populations. Perceived stress scale (mostly the 4-item version, i.e., the one with poorest psychometric property) has been used in studies conducted to assess stress among LGBT population [22–27]. However, no study seemed to be concerned about performing factor analysis or validation of PSS on LGBT population. Risk of drawing faulty conclusion from poorly- or non-validated tool is too great. A tool developed in one culture may measure completely different construct for other cultures especially for a minority group (see [28]). The present study was aimed at validation along with exploration and comparison of factor structure of PSS 10 among LGBT population in Bangladesh.

A recent estimate suggests the size of MSM (men who have sex with men) population to be between 21,833 to 110,581 and the transgender population to be between 4504 to 8882 in Bangladesh [29]. The size of lesbian and bisexual population in Bangladesh is still unknown. Although commonly coined together as LGBT, the four sexual minority population have differences in terms of their psychosocial circumstances in Bangladesh. LGBT population, except for the transgender group, are largely hidden in Bangladesh due to lack of socio-cultural and legal acceptance. Transgender people commonly known as Hijras in Bangladesh usually live and roam around in well connected groups segregated from mainstream society with a distinct culture of their own (see [30]). They are easily identifiable with their distinctive appearance (feminine dress up with masculine facial features, voice tone, and body built) and activities (claiming and collecting money from the shops; singing, dancing and claiming money from parents when babies are born). Among the other members of LBGT, a portion of the gays have feminine behavior pattern which often leads to their identification and subsequent victimization. The remaining two groups i.e., lesbians and bisexuals, kept themselves unidentified and face lesser daily hassles associated with disclosed identity. While the often married bisexuals face difficulties in maintaining dual relation, the lesbians face stress when their parents apply force to marry them off to men. These differences necessitate the need to check whether or not PSS10 have comparable psychometric properties among the lesbian, gay, bisexual and transgender population.

Published data on stress and mental health of LGBT population in Bangladesh are limited. However, different studies equivocally reported presence of extreme stressors in their life [30–32]. Utility of a validated measure of stress for LGBT population in Bangladesh would be enormous.

## Methods

### Participants

This cross sectional study employed a combination of sampling strategies in selecting participants. To ensure representativeness, five divisional districts of Bangladesh namely, Dhaka, Khulna, Mymensing, Rangpur and Sylhet were randomly selected. Hard to reach LGBT participants were then selected from these districts using convenience sampling. Lesbian participants were only found in Dhaka and Khulna district. Among the 297 adult participants there were 34 lesbian, 85 gay, 87 bisexual and 91 transgender individuals. Checking for multivariate outlier resulted removal of one lesbian participant reducing the number of lesbian participants into 33. Educational attainment of the participants varied from no formal education to graduation (Table 1).

**Table 1:** Socio-demographic characteristics of participants

Variable	Lesbian *N* = 33	Gay *N* = 85	Bisexual *N* = 87	Transgender *N* = 91
Educational attainment n(%)				
No formal education	2 (6.06)	7 (8.43)	9 (10.47)	22 (24.44)
Primary	11 (33.33)	18 (21.69)	28 (32.56)	36 (40.00)
Secondary	4 (12.12)	21 (25.30)	17 (19.77)	22 (24.44)
Higher Secondary	6 (18.18)	24 (28.92)	18 (20.93)	6 (6.67)
Undergraduate	9 (27.27)	12 (14.46)	13 (15.12)	3 (3.33)
Graduate	1 (3.03)	1 (1.20)	1 (1.16)	1 (1.11)
Source of finance n(%)				
Service	13 (39.39)	14 (16.47)	18 (20.69)	1 (1.10)
Business	1 (3.03)	3 (3.53)	18 (20.69)	0 (0.00)
Family	6 (18.18)	14 (16.47)	13 (14.94)	0 (0.00)
Multiple	4 (12.12)	42 (49.41)	23 (26.44)	51 (56.04)
Others	9 (27.27)	12 (14.12)	15 (17.24)	39 (42.86)
Age M(SD)	28.94 (8.07)	26.84 (8.09)	26.87 (6.88)	30.94 (11.99)
Income M(SD)^a^	15.59(13.39)	10.92(8.26)	15.01(11.57)	12.45(8.63)

^a^Monthly income in thousand Taka

### Instruments

#### Perceived stress scale [PSS 10, 2]

As recommended by Cohen and Williamson [13], PSS 10 (i.e., the 10-item version) was used in this study for its superior psychometric properties over the two other versions (PSS 14 & PSS 4). PSS 10 demonstrated good internal consistency where Cronbach’s alpha ranged from 0.71–0.91 among different populations [7, 33]. Test-retest reliability of the scale was reported with correlation value *r* > 0.70 in different studies (see [34]). Construct validity of PSS 10 has been tested most commonly with concurrent method using diverse tools such as depression scale, anxiety scale, state-trait anxiety scale, impact of event scale, general health questionnaire (GHQ) and life event scale indicating moderate to strong correlation [13, 34]. Unavailability of suitable gold standard resulted in scarce reports on criterion validity for PSS 10 [34]. Bengali version of PSS 10 (see Additional file 1) used in this study was translated by Ziaul Islam and is available at Sheldon Cohen’s Laboratory for the Study of Stress [35], however, no published data on validity or reliability of this Bengali PSS 10 is available.

#### Self reporting questionnaire [SRQ 20, 36]

SRQ is widely used for assessing and screening overall psychological morbidity. With a value ≥8 considered as the cutoff [36], its score may range from 0 to 20 where higher score is indicative of higher problem. Sensitivity and specificity of SRQ 20 ranged from 73% - 83% and 72% - 85% respectively [36]. High Cronbach’s alpha (>0.80) reported in different studies proves SRQ 20 to be internally consistent [37, 38]. SRQ 20 has been standardized in several countries including Bangladesh and is widely used as a research tool [39–41].

### Procedures

This study was carried out as part of a bigger study on exploring mental health state of LGBT population in Bangladesh. Original study questionnaire contained 274 variable-response items. Thirteen data collectors with honors degree in psychology were provided two-day rigorous training to sensitize them about LGBT population and to reduce inter-interviewer variation in data collection. Organizations working with LGBT population in the selected five districts were approached for recruiting study participants. Verbal as well as written explanatory statements were used to inform participants about the nature of the study, risks and benefits associated with participation, confidentiality and anonymity in publication. Verbal informed consent was taken instead of written consent for ensuring identity protection and thus to avoid any potential legal implications for the participants (see [42]). It may be noted that homosexuality is legally prohibited in Bangladesh [43, 44]. Nominal compensation (Tk.250, approximately US$3) was provided for their wage loss and cost of transport.

## Results

### Factor analysis

Sampling adequacy for factor analysis was tested separately for four groups of participants. Kaiser-Meyer-Olkin (KMO) scores ranged from 0.67 to 0.72 indicating mediocre to middling level of sampling adequacy [45] which were above the recommended value of 0.6 [46]. Bartlett’s test of sphericity also indicated suitability of factor analysis for each group (χ^2^ ranged from 105.68 to 229.44, all significant at *p* ≤ 0.001). Shared variance was indicated by communalities where values of all items for all four populations were above 0.3 adding final evidence that factor analysis can be carried out on these samples with the 10 items of PSS.

Exploratory factor analysis (EFA) using principle component analysis was carried out. Component correlation matrix suggested the factors to be uncorrelated (|r| = 0.026 to 0.203) and hence varimax rotation was chosen. Inspection of eigenvalues, screeplots and parallel analyses [47] suggested a two-factor solution. Items under each factor had adequate loading (ranged from 0.435 to 0.877) supporting retention of all items of PSS 10 (Table 2).

**Table 2:** Factor structure of PSS 10 for LGBT population

Item no.	Lesbian		Gay		Bisexual		Transgender	
	F 1	F 2	F 1	F 2	F 1	F 2	F 1	F 2
1	0.54	0.08	0.72	−0.02	0.74	0.13	0.74	0.01
2	0.86	−0.11	0.67	−0.09	0.74	0.20	0.64	0.01
3	0.80	0.12	0.88	−0.06	0.56	0.02	0.78	−0.06
4	0.09	0.60	0.02	0.77	0.22	0.72	0.12	0.72
5	0.22	0.68	0.21	0.65	0.16	0.70	−0.08	0.63
6	0.48	−0.28	0.52	0.23	0.43	0.08	0.50	−0.13
7	−0.01	0.64	−0.09	0.71	0.14	0.60	−0.07	0.51
8	−0.15	0.61	−0.32	0.69	−0.25	0.63	−0.23	0.66
9	0.86	0.12	0.71	−0.07	0.63	−0.13	0.51	−0.34
10	0.83	0.10	0.56	−0.06	0.62	0.38	0.66	−0.07
Eigen value	*3.40*	*1.74*	*2.99*	*2.06*	*2.52*	*1.98*	*2.59*	*1.76*
% variance	*33.99*	*17.45*	*29.90*	*20.64*	*25.21*	*19.80*	*25.95*	*17.60*

*F1* Factor 1, *F2* Factor 2

Confirmatory factor analysis (CFA) was performed in AMOS 18 [48] to test goodness of fit of the two-factor structure extracted from EFA (Table 3). Adequacy of model fit was assessed with multiple indices including Chi-squire (χ^2^), ratio of Chi-squire to df (χ^2^/df), root mean square error of approximation (RMSEA), comparative fit index (CFI), Tucker-Lewis index (TLI), and standardized root mean squire residual (SRMR). Criteria for model fit were, χ^2^ with *p* ≥ 0.01, χ^2^/df ≤ 2, RMSEA ≤0.06, CFI ≥ 0.95, TLI ≥ 0.95, and SRMR ≤0.08 [49, 50].

**Table 3:** Goodness of fit indices for two-factor model of PSS 10 on LGBT population

	χ^2^	df	p	χ^2^/df	RMSEA (CI)	CFI	TLI	SRMR
Lesbian	36.413	34	0.357	1.071	0.056 (0.00–0.14)	0.968	0.958	0.105
Gay	62.464	34	0.002	1.837	0.100 (0.06–0.14)	0.855	0.808	0.094
Bisexual	43.629	34	0.125	1.283	0.057 (0.00–0.10)	0.927	0.904	0.078
Transgender	34.657	34	0.436	1.019	0.015 (0.00–0.08)	0.994	0.992	0.067

The indices suggest model fit for lesbian, bisexual and transgender population. Model fit for gay population was indicated only on χ^2^/df criteria.

### Reliability

Internal consistency reliability of PSS 10 full scale and the Factors 1 was indicated for lesbian, gay and bisexual population with Cronbach’s alpha values (Table 4). However, poor Cronbach’s alpha on full scale (0.485) was found for transgender population indicating lack of internal consistency for this group. In general, Factor 2 had poorer internal consistency compared to Factor 1.

**Table 4:** Internal consistency reliability and convergent validity of PSS10 and its two factors

	Internal consistency (Cronbach’s alpha)			Inter-correlation (r)	Convergent validity (Correlation with SRQ)		
	Full scale	F1	F2	F1 & F2	Full scale	F1	F2
Lesbian	0.713	0.832	0.536	0.065	0.833*	0.818*	0.294
Gay	0.624	0.772	0.671	−0.112	0.530*	0.489*	0.163
Bisexual	0.712	0.714	0.597	0.270**	0.646*	0.652*	0.315*
Transgender	0.485	0.730	0.539	−0.233**	0.519*	0.467*	0.122

*F1* Factor 1, *F2* Factor 2; *p ≤ 0.01, **p ≤ 0.05

### Validity

Moderate to strong correlation between scores on SRQ 20 and PSS 10 as well as Factor 1 for the LGBT population (Pearson r ranged from 0.467 to 0.833; all significant at *p* ≤ 0.001) indicates convergent validity for Bengali version of PSS 10 among LGBT population (Table 4). Poor correlation was observed between scores on SRQ and Factor 2 indicating lack of convergence except for bisexual group.

## Discussion

The present study assessed factor structure, reliability and validity of PSS 10 among lesbian, gay, bisexual and transgender population in Bangladesh. The findings revealed a two-factor structure of the scale suitable for all four populations, which is consistent with findings from the original study [13] as well as other studies conducted in Asia, Africa, Europe, and Americas on PSS 10 factor structure [5, 6, 11, 15, 51]. In three instances, (item 8 for gay, item 9 for transgender, and item 10 for bisexual population) items demonstrated loading at 0.30s range on the alternative factor, however, this is not uncommon in the PSS 10 literature (see [7]). The dominant loading was taken into consideration in deciding factor structure in such cases. The amount of total variance explained by the two factors ranged from 43.55% - 51.44%, which is consistent with published studies on PSS 10 [7, 13]. In line with Örücü and Demir [11] the two factors in the Bengali PSS 10 can be termed as ‘perceived helplessness’ and ‘perceived self-efficacy’.

Multiple fit indices consulted during CFA indicated good fit of the two-factor model among the lesbian, bisexual and transgender population. Model fit for gay population was only supported by χ^2^/df criterion. Additionally, SRMR value (0.094) can be considered as indicative of acceptable fit of the model for gay population if a less stringent criterion (SRMR ≤0.10) is used (see [49]). Modification of the model could lead to better fit, however, no modification was performed to maintain comparability of the model among the four populations. One challenge in interpreting these model fits is the small sized samples used in analyses especially for the lesbian group. As RMSEA and SRMR values are inflated with smaller sized samples, it can be assumed that larger samples would indicate better fit. However, CFI and TLI indices used here are claimed to be less affected by sample size and hence can be considered reliable indicators of fit in the present study.

PSS 10 was internally consistent for lesbian, gay and bisexual population. Poor Cronbach’s alpha was found for transgender population. Additional analysis revealed negative correlation (− 0.233) between the two factors in transgender population. Lack of internal consistency in transgender population can therefore be interpreted as result of distinctiveness of the two factors. It should be noted here that all the fit indices suggested good fit of the two-factor structure of PSS for the transgender population (Table 3). Internal consistency for Factor 1 was sufficient for all groups while poor Cronbach’s alpha (0.536–0.671) was revealed for Factor 2. It should be noted that Cronbach’s alpha is heavily influenced by the number of items in the instrument. Lower alpha on Factor 2 can be caused by having only four items in the factor. Inter-correlation between the two factors for two groups (Lesbian and Gay) was very low and non-significant which is rarely found in PSS 10 literature [14] while for the two other (Bisexual and Transgender) it was at moderate level and significant (one correlated positively and the other negatively; see Table 4). These findings can be indicative of differences in response pattern and therefore in perception of stress for the four sub-groups of LGBT.

Due to sensitive nature of the topic, no names, addresses or other identification data were collected from the participants. Anonymity was also crucial for averting putative legal implications associated with identification of the participants [42, 43]. This anonymity prevented planning for assessing test-retest reliability of the PSS 10 for LGBT population. Testing for criterion validity could not be carried out due to lack of alternative gold standard for assessing perceived stress. Few studies claimed criterion validity of PSS 10, using mental component of SF-36 as the criterion measure [33]. However, as the two tools measure different constructs, it is likely that correlation of PSS 10 and SF 36 mental component is actually indicative of construct validity for PSS 10 instead of criterion validity.

The findings indicated construct validity of Bengali PSS 10 through convergent method. This study used SRQ 20 for validation instead of common practice of using GHQ, depression inventory, anxiety inventory or life event scales [34]. SRQ 20 seemed more suitable as it is known to directly measure overall psychological morbidity and has also been validated in Bangladeshi community sample [52]. High correlation was found between PSS 10 and SRQ 20 scores for full scale and Factor 1, while poor correlation was found for Factor 2.

Poor psychometric properties for Factor 2 have been reported in previous studies [4, 5, 14] however, these were not so drastic as in the case of present study. Psychometric properties for Factor 2 i.e., ‘perceived self-efficacy’ part of PSS 10 suggest against possibility of using the two factors of scale as two subscales for LGBT population in Bangladesh.

## Conclusions

These findings reinforced the robustness of PSS 10 by providing evidence of comparable factor structure, validity and reliability among lesbian, gay, bisexual and transgender population. Thus it suggests usability of PSS 10 among sexual minority population that is LGBT. Evidence of validity and reliability of PSS in these populations are likely to increase confident use of the 10-item perceived stress scale as a measure of stress appraisal among LGBT population worldwide and in Bangladesh. However, factor level analysis indicated poorer psychometric properties for Factor 2, restricting usability of Factor 2 as a sub-scale in assessing perceived stress among LGBT. Moreover, small sample size for the LGBT populations (especially for the Lesbian) raised concern regarding robustness of the estimates, requiring readers to uses caution in interpreting the findings.

## Additional file

Additional file 1: Bengali Perceived Stress Scale (PSS 10); File name: Bengali PSS. (PDF 493 kb)

## Abbreviations

CFA: Confirmatory factor analysis
CFI: Comparative fit index
EFA: Exploratory factor analysis
GHQ: General health questionnaire
LGBT: Lesbian, gay, bisexual and transgender
MSM: Men who have sex with men
PSS: Perceived Stress Scale
RMSEA: Root mean square error of approximation
SRMR: Standardized root mean squire residual
SRQ: Self Reporting Questionnaire
TLI: Tucker-Lewis index
