# Supplementary material for: Validation of Bengali perceived stress scale among LGBT population
Source: BMC Psychiatry. 2017 Aug 29;17:314. doi: 10.1186/s12888-017-1482-0 (PMC5576282; doi:10.1186/s12888-017-1482-0)
Supplement: Additional file 1: — Bengali Perceived Stress Scale (PSS 10); File name: Bengali PSS. (PDF 493 kb) [file 12888_2017_1482_MOESM1_ESM.pdf]

Perceived Stress Scale 10 Item

নির্দেশনাঃ

নিচের প্রশ্নে উল্লিখিত অনুভুতি এবং ভাবনাগুলো গত **এক মাসে** আপনার মধ্যে কি পরিমানে ঘটেছে তা ডান পাশের পাচটির মধ্যে উপযুক্ত ঘরে টিক (X) চিহ্ন দিয়ে নির্দেশ করুন।

		কখনই না	অনেকাংশে না	মাঝে মাঝে	প্রায়শই	ঘন ঘন
১	গত এক মাসে অনাকাঙ্ক্ষিত কোন ঘটনার জন্য আপনি কতটুকু বিপর্যস্ত ছিলেন ?	০	১	২	৩	৪
২	গত এক মাসে আপনি কতটুকু অনুভব করতে পেরেছিলেন যে আপনার জীবনের গুরুত্বপূর্ণ ঘটনাগুলো আপনি নিয়ন্ত্রণ করতে পারছেন না ?	০	১	২	৩	৪
৩	গত এক মাসে আপনি কতটুকু ঘাবড়ে যাওয়া এবং চাপ অনুভব করেছিলেন ?	০	১	২	৩	৪
৪	গত এক মাসে আপনার ব্যক্তিগত সমস্যাগুলো নিয়ন্ত্রনের ক্ষেত্রে আপনি কতটুকু আত্মবিশ্বাসী ছিলেন?	০	১	২	৩	৪
৫	গত এক মাসে আপনি কতটুকু অনুভব করেছিলেন যে চলমান ঘটনাগুলো আপনার অনুকুলে যাচ্ছে ?	০	১	২	৩	৪
৬	গত এক মাসে আপনি কতটুকু অনুভব করেছিলেন যে আপনার যা করণীয় তা আপনি করতে পারেন নি ?	০	১	২	৩	৪
৭	গত এক মাসে আপনি আপনার জীবনের বিরক্তি/ তিক্ততা কতটুকু নিয়ন্ত্রন করতে পেরেছিলেন ?	০	১	২	৩	৪
৮	গত এক মাসে আপনি কতটুকু অনুভব করেছিলেন যে আপনি সবকিছুর ঊর্ধ্বে ? (আপনার প্রাধান্য বেশি)	০	১	২	৩	৪
৯	গত এক মাসে নিয়ন্ত্রনের বাহিরে যাওয়া কোন ঘটনার জন্য আপনি কতটুকু ক্রোধান্বিত হয়েছিলেন ?	০	১	২	৩	৪
১০	গত এক মাসে আপনি কতটুকু অনুভব করেছিলেন যে জীবনের জটিলতাগুলো এতই বড় যে আপনি তা অতিক্রম করতে পারবেন না ?	০	১	২	৩	৪

Source:
This scale was translated by Professor Dr. Md. Ziaul Islam, and is available at Sheldon Cohen's Laboratory for the Study of Stress at the following web address,
http://www.psy.cmu.edu/~scohen/Perceived_Stress_Scale_Bengali_translation.pdf
